# Early Pandemic Influenza (2009 H1N1) in Ho Chi Minh City, Vietnam: A Clinical Virological and Epidemiological Analysis

**DOI:** 10.1371/journal.pmed.1000277

**Published:** 2010-05-18

**Authors:** Tran Tinh Hien, Maciej F. Boni, Juliet E. Bryant, Tran Thuy Ngan, Marcel Wolbers, Tran Dang Nguyen, Nguyen Thanh Truong, Nguyen Thi Dung, Do Quang Ha, Vo Minh Hien, Tran Tan Thanh, Le Nguyen Truc Nhu, Le Thi Tam Uyen, Pham Thi Nhien, Nguyen Tran Chinh, Nguyen Van Vinh Chau, Jeremy Farrar, H. Rogier van Doorn

**Affiliations:** 1 Oxford University Clinical Research Unit, Wellcome Trust Major Overseas Program, Hospital for Tropical Diseases, Ho Chi Minh City, Vietnam; 2 Hospital for Tropical Diseases, Ho Chi Minh City, Vietnam; 3 Southeast Asian Infectious Diseases Clinical Research Network (SEAICRN, Jakarta, Indonesia; 4 MRC Centre for Genomics and Global Health, University of Oxford, Oxford, United Kingdom; 5 Centre for Tropical Medicine, Nuffield Department of Clinical Medicine, University of Oxford, Centre for Clinical Vaccinology and Tropical Medicine, Oxford, United Kingdom; Harvard School of Public Health, United States of America

## Abstract

**Background:**

To date, little is known about the initial spread and response to the 2009 pandemic of novel influenza A (“2009 H1N1”) in tropical countries. Here, we analyse the early progression of the epidemic from 26 May 2009 until the establishment of community transmission in the second half of July 2009 in Ho Chi Minh City (HCMC), Vietnam. In addition, we present detailed systematic viral clearance data on 292 isolated and treated patients and the first three cases of selection of resistant virus during treatment in Vietnam.

**Methods and Findings:**

Data sources included all available health reports from the Ministry of Health and relevant health authorities as well as clinical and laboratory data from the first confirmed cases isolated at the Hospital for Tropical Diseases in HCMC. Extensive reverse transcription (RT)-PCR diagnostics on serial samples, viral culture, neuraminidase-inhibition testing, and sequencing were performed on a subset of 2009 H1N1 confirmed cases. Virological (PCR status, shedding) and epidemiological (incidence, isolation, discharge) data were combined to reconstruct the initial outbreak and the establishment of community transmission. From 27 April to 24 July 2009, approximately 760,000 passengers who entered HCMC on international flights were screened at the airport by a body temperature scan and symptom questionnaire. Approximately 0.15% of incoming passengers were intercepted, 200 of whom tested positive for 2009 H1N1 by RT-PCR. An additional 121 out of 169 nontravelers tested positive after self-reporting or contact tracing. These 321 patients spent 79% of their PCR-positive days in isolation; 60% of PCR-positive days were spent treated and in isolation. Influenza-like illness was noted in 61% of patients and no patients experienced pneumonia or severe outcomes. Viral clearance times were similar among patient groups with differing time intervals from illness onset to treatment, with estimated median clearance times between 2.6 and 2.8 d post-treatment for illness-to-treatment intervals of 1–4 d, and 2.0 d (95% confidence interval 1.5–2.5) when treatment was started on the first day of illness.

**Conclusions:**

The patients described here represent a cross-section of infected individuals that were identified by temperature screening and symptom questionnaires at the airport, as well as mildly symptomatic to moderately ill patients who self-reported to hospitals. Data are observational and, although they are suggestive, it is not possible to be certain whether the containment efforts delayed community transmission in Vietnam. Viral clearance data assessed by RT-PCR showed a rapid therapeutic response to oseltamivir.

*Please see later in the article for the Editors' Summary*

## Abstract 2

Rogier van Doorn and colleagues analyze the initial outbreak, attempts at containment, and establishment of community transmission of pandemic H1N1 influenza in Ho Chi Minh City, Vietnam.

## Editors' Summary

**Background:**

Every year, millions of people catch influenza—a viral infection of the airways—and about half a million people die as a result. These yearly seasonal epidemics occur because small but frequent changes in the influenza virus mean that the immune response produced by infection with one year's virus provides only partial protection against the next year's virus. Sometimes, however, a very different influenza virus emerges to which people have virtually no immunity. Such viruses can start global epidemics (pandemics) and can kill millions of people. Consequently, when the first case of influenza caused by a new virus called pandemic A/H1N1 2009 (2009 H1N1, swine flu) occurred in March 2009 in Mexico, alarm bells rang. National and international public health agencies quickly issued advice about how the public could help to control the spread of the virus and, as the virus spread, some countries banned flights from affected regions and instigated screening for influenza-like illness at airports. However, despite everyone's efforts, the virus spread rapidly and on June 11, 2009 the World Health Organization (WHO) declared that an influenza pandemic was underway.

**Why Was This Study Done?:**

To date, little is known about the spread of and response to 2009 H1N1 in tropical countries. In this study, therefore, the researchers investigate the early progression of the 2009 H1N1 pandemic in Ho Chi Minh City, Vietnam, and the treatment of infected patients. On April 27, 2009, when WHO announced that human-to-human transmission of 2009 H1N1 was occurring, the Vietnamese Ministry of Health mandated airport body temperature scans and symptom questionnaire screening of travelers arriving in Vietnam's international airports. Suspected cases were immediately transferred to in-hospital isolation, screened for virus using a sensitive test called PCR, and treated with the anti-influenza drug oseltamivir if positive. The first case of 2009 H1N1 infection in Vietnam was reported on May 31, 2009 in a student who had returned from the US on May 26, 2009, and, despite these efforts to contain the infection, by the second half of July the virus was circulating in Ho Chi Minh City (community transmission).

**What Did the Researchers Do and Find?:**

The researchers used reports from the Ministry of Health and relevant health authorities and clinical and laboratory data for people infected with 2009 H1N1 and isolated in hospital to reconstruct the initial outbreak and the establishment of community transmission in Ho Chi Minh City. Between April 27 and July 24 2009, three-quarters of a million passengers arriving in the city on international flights were screened at the airport. 200 passenger tested positive for 2009 H1N1 as did 121 nontravelers who were identified during this period after self-reporting illness or through contact tracing. The infected individuals spent 79% of the days when they tested positive for 2009 H1N1 by PCR (days when they were infectious) in isolation; 60% of their PCR-positive days were spent in isolation and treatment. Importantly, travelers and nontravelers spent 10% and 42.2%, respectively, of their potentially infectious time in the community. None of the patients became severely ill but 61% experienced an influenza-like illness. Finally, the average time from starting treatment to clearance of the virus was between 2.6 and 2.8 days for patients who began treatment 1 to 4 days after becoming ill; for those who started treatment on the first day of illness, the average virus clearance time was 2.0 days.

**What Do These Findings Mean?:**

These findings, although limited by missing data, suggest that the strict containment measures introduced early in the 2009 H1N1 pandemic in Ho Chi Minh City may have reduced the circulation of infected people in the community. This reduction in circulation might have delayed the onset of community transmission, suggest the researchers, but because the study was observational, this possibility cannot be proven. However, importantly, these findings show that the containment measures were unable to prevent the eventual establishment of pandemic influenza in Vietnam, presumably because many imported cases were not detected by airport screening. Finally, these findings suggest that in Vietnam, as in other countries, 2009 H1N1 causes a mild disease and that this disease responds quickly to treatment with oseltamivir whenever treatment is started in relation to the onset of illness.

**Additional Information:**

Please access these Web sites via the online version of this summary at http://dx.doi.org/10.1371/journal.pmed.1000277.

- The US Centers for Disease Control and Prevention provides information about influenza for patients and professionals, including specific information on H1N1 influenza and how to prevent its spread
- Flu.gov, a US government website, provides information on H1N1, avian, and pandemic influenza
- The World Health Organization provides information on seasonal influenza and has detailed information on H1N1 influenza (in several languages); the WHO Representative Office in Vietnam provides an overview of the current 2009 H1N1 situation in Vietnam
- The UK Health Protection Agency provides information on pandemic influenza and on H1N1 influenza
- Wikipedia has a timeline of the 2009 H1N1 pandemic (note that Wikipedia is a free online encyclopedia that anyone can edit; available in several languages)

## Introduction

Vietnam reported its first case of infection with 2009 pandemic influenza virus A (H1N1) on 31 May 2009, in a Vietnamese student returning from Wisconsin (United States) who had arrived at the international airport of Ho Chi Minh City (HCMC) on 26 May 2009. 12 d later on 12 June, Hanoi reported its first cases. When the World Health Organization (WHO) declared pandemic phase 4 on 27 April 2009, the Vietnamese Ministry of Health mandated airport body temperature scans and symptom questionnaire screening of arriving international travelers and in-hospital isolation of suspected cases, ensuring that symptomatic passengers were intercepted, transferred to a hospital, screened by reverse transcription (RT)-PCR, and treated if positive. At that time, the Pasteur Institute and the Hospital for Tropical Diseases (HTD) were the only two laboratories performing the WHO/US Centers for Disease Control (USCDC) influenza virus A RT-PCR in HCMC. Both labs received specific primers for the novel virus by the second week of May, and after that point the Pasteur Institute provided formal national diagnostic confirmation of 2009 H1N1 infection. After 29 May, HTD served as the main referral centre for confirmed 2009 pandemic influenza treatment within the city, including cases identified from airport interceptions and community outbreaks. Passengers testing positive were transferred to HTD, where they were isolated, treated with oseltamivir, and followed up clinically for at least 5 d and until RT-PCR negative.

Here we review available virological and epidemiological data of pandemic influenza importation and transmission in HCMC from 26 May to 24 July 2009. During this period, HTD had responsibility for clinical follow-up of cases diagnosed by the Pasteur Institute, and for primary diagnosis of suspected patients reporting to our outpatient clinics or transferred from other hospitals/clinics. We describe the epidemiological, clinical, and viral clearance characteristics of cases of 2009 pandemic influenza during this early phase of containment and the establishment of community transmission, and discuss implications and forecasts for the progression of the outbreak.

For brevity, hereafter we use the term “2009 H1N1” to refer to the virus and the disease caused by the novel influenza virus A/H1N1/2009 that was identified in Mexico and the United States in late April 2009. Data on clearance of viral RNA and viable virus were reported previously on ProMED-mail [1]–[3].

## Methods

### Data Sources

**Figure 1:**
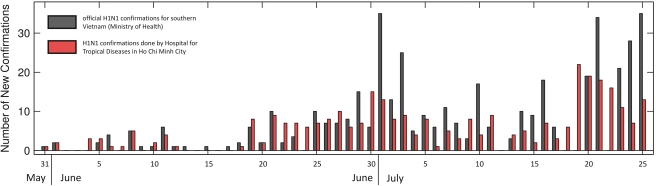
New cases as reported by the Ministry of Health and Hospital for Tropical Diseases. Day-by-day comparison of official H1N1 confirmations in southern Vietnam as reported by the Ministry of Health in Vietnam (dark gray bars) and by the surveillance and diagnostics laboratories of HTD/Oxford University Clinical Research Unit (OUCRU) and the HCMC Health Services (red bars) during the initial epidemic phase in HCMC. Overall, 321 H1N1 confirmations were captured by HTD, OUCRU, and the HCMC Health Services, out of a total of 424 reported for southern Vietnam during the period from 31 May to 25 July 2009. HTD confirmations are a subset of Ministry of Health confirmations; reporting dates for individual cases can differ.

Data sources included the first 30 reports on the 2009 H1N1 response from the Health Services of HCMC [4], dating from 10 May to 9 July 2009, as well as comprehensive clinical and diagnostic information for the first 300 2009 H1N1-confirmed patients admitted to HTD, between 29 May and 25 July 2009. Eight of 300 patients were excluded from clinical and virological analysis because of missing test results. The Health Services reports provided information regarding (i) the daily numbers of incoming air travel passengers arriving at HCMC airport; (ii) numbers of persons isolated; (iii) diagnostic results and isolation status of intercepted travelers; and (iv) reporting and diagnostic confirmation of 2009 H1N1 from patients voluntarily presenting to all other health care facilities in the city. Data from HTD diagnostics and HCMC Health Services reports were combined into a final dataset comprising 321 PCR-positive individuals and 298 PCR-negative individuals. Vietnam's Ministry of Health reported 424 molecular confirmations of 2009 H1N1 between 31 May and 25 July 2009 in southern Vietnam; our dataset represents 321 (76%) of these cases, making it representative of the total known case burden of 2009 H1N1 in southern Vietnam during these 8 wk (Figure 1).

### Patient Samples and Clinical Data

Upon molecular confirmation of 2009 H1N1, all patients were admitted to the HTD isolation ward and treated with 75 mg oseltamivir twice a day for 5 d. Initial data on symptoms were collected, chest X-rays and ECGs performed, blood specimens collected for hematology and biochemistry, and respiratory specimens collected for RT-PCR. Patients admitted between 29 May and 29 June 2009 were tested daily by RT-PCR until negative. From 30 June to 16 July 2009, the sampling schedule for RT-PCR diagnostics was modified to reduce the workload of the hospital laboratory; respiratory sampling for RT-PCR was limited to the day of admission and day 5 after admission. Starting on 17 July 2009, the sampling schedule was again modified to include day 3 respiratory specimens, with the objective of facilitating early discharge of PCR-negative patients. Patients still positive after 5 d received an additional 5-d course of oseltamivir (*n* = 18) and were sampled daily until PCR-negative. One patient received 5 d of zanamivir because of PCR positivity after 10 d of oseltamivir. The vast majority of respiratory specimens were combined nasal and throat swabs in viral transport medium, however, small numbers of throat swabs, nasopharyngeal aspirates, sputum, and rectal swabs were also received and processed.

### Molecular Diagnostics

Real-time RT-PCR diagnostics were conducted using protocols designed and distributed by WHO/USCDC for detection of influenza A (InfVA) and swine influenza A (swInfVA) viruses [5]. An additional in-house protocol (swH1) was used to confirm 2009 H1N1 using oligonucleotides designed on the basis of the first eight available 2009 H1N1 sequences, targeting the haemagglutinin (HA) gene (forward, AGC TAA RAA ACA ATG CCA ARG AA; reverse, TGC ACG TGT YAT CRC ATT TG; probe, 6-Fam-TGG AAA YGG CTG CTT TGA ATT YTA YC-BHQ). Reaction mix and thermocycling protocol for this PCR were the same as for the WHO/USCDC assays. These in-house primer sequences provided an improved match with circulating 2009 H1N1 isolates compared to those initially distributed by WHO/USCDC (unpublished data). Analytical sensitivities of the InfVA, swInfVA, and swH1 PCRs were in the order of magnitude of 5, 50, and 50 copies/assay, respectively. Specimens collected were processed for all three targets. Viral clearance analysis was performed using only the universal InfVA assay, as this was found to be the most sensitive of the three PCRs (unpublished data).

Selected 2009 H1N1-positive samples with sufficient viral load (Ct value<38) were screened for the presence of the oseltamivir resistance–associated mutation H275Y in the neuraminidase (NA) gene by an in-house real-time RT-PCR protocol using two reverse primers with a 3′ locked nucleic acid (LNA) residue that either hybridizes with the wild-type or mutant allele and a TaqMan probe (forward, TAGAAAAGGGAAAGATAGTCAAA; reverse Wt, ACAGGAGCATTCCTCATAGTG; reverse M, ACAGGAGCATTCCTCATAGTA; probe, FAM-CAGTCGAAATGAATGCCCCTAATTA-BHQ1, unpublished data), followed by confirmatory partial sequencing of NA directly on clinical specimens or on isolated virus using primers described previously [6]. Analytical sensitivity of this PCR was 100 copies/assay. Phenotypic screening for oseltamivir resistance was also conducted on selected virus isolates, using the fluorogenic substrate MUNANA as previously described [7],[8].

Virus culture was conducted using MDCK-Siat1 cells (kindly made available by Mikhail Matrosovich) [9], in a six-well plate format with a maximum of three passage attempts per specimen.

### Duration of PCR Positivity

Because not all patients were tested daily, each patient's duration of PCR positivity corresponds to an interval-censored observation defined by that patient's last positive PCR result and the first negative PCR result (minus one day). We used a parametric logistic survival model and maximum likelihood (ML) estimation to estimate the distribution of a patients' duration of PCR positivity. The logistic model was chosen as it provided a better fit, in terms of the Akaike information criterion, than Weibull, lognormal, or log-logistic distributions.

### Imputation of Missing Data

Epidemiological analysis was carried out on a combined dataset of 321 PCR-positive patients representing the symptomatic cases diagnosed in HCMC between 26 May and 24 July 2009. For each patient, day of hospital admission and recent travel status were known, making it possible to infer if a patient was infected abroad or in Vietnam. Other relevant data for these patients were date of arrival into HCMC, date of admission to hospital, date of illness onset, date of treatment commencement, reported date of last positive PCR, and first negative PCR. Missing data included 145 dates of arrival at HCMC International Airport, 29 dates of illness onset, 29 dates of treatment commencement, 41 dates of reported first negative PCR result, 29 dates of reported last positive PCR result. It appears most plausible to us that missing data are due to purely administrative omissions (a failure to record the respective information in the patients' file), i.e., these data are missing completely at random (MCAR). Dates of arrival, illness onset, and treatment commencement were imputed as follows. Date of arrival was imputed on the basis of the empirical distribution of the arrival-to-admission time interval for the travelers for whom it was known. Date of illness onset was imputed on the basis of observed illness-to-admission interval for travelers and residents separately. Date of treatment commencement was imputed on the basis of the admission-to-treatment interval. Finally, we imputed the true duration of PCR positivity (i.e., assuming ideal daily PCR tests) on the basis of the maximum-likelihood inferred logistic distribution conditioned on the patient's reported last positive PCR result, first negative PCR result (if available), and the interval of time between illness onset and treatment initiation. We used multiple imputation, and results are reported as 95% ranges (i.e., 2.5%–97.5% quantiles from imputed data [QID]) on the basis of 100 randomly imputed datasets.

## Results

### Epidemiology

From 27 April through 9 July 2009, a total of 630,778 passengers entered HCMC on international flights; 361,143 of these coming from countries, either directly or transiting, that had confirmed cases of 2009 H1N1. Of these travelers, 967 (0.15%) were intercepted by airport screening procedures—body temperature scan and symptom questionnaire—as febrile and potentially infected. Individual patient diagnostic data are available for 450 passengers intercepted between 27 April and 24 July 2009. Of these 450, 200 (44%) tested positive by RT-PCR for 2009 H1N1. An additional 169 residents of HCMC—defined here as individuals who were neither intercepted at the airport nor followed up a few days after arriving in Vietnam from abroad—were tested after contact tracing or self-reporting to city hospitals with influenza-like symptoms, and 121 (72%) of these were PCR-positive for 2009 H1N1. Table 1 presents a summary of these data; the two datasets do not overlap perfectly as the last available HCMC Health Services report was dated 9 July 2009.

**Table 1:** Characteristics of study participants.

Travelers and HCMC residents from whom data were used in this study	27 April–9 July 2009^a^	27 April–24 July 2009
**Number of passengers arriving at Tan Son Nhat International Airport in HCMC**		
**From all countries**	630,778	about 760,000^b^
**From epidemic countries**	361,143	Unknown^c^
**Number of arriving passengers suspected of influenza, isolated, and monitored**	967	Unknown
**Number of arriving passengers subsequently determined to be PCR-positive for 2009 H1N1 at HTD laboratories in HCMC**	153–166^d^	200^e^
**Number of residents (nontravelers) admitted to hospital with symptoms and tested PCR-positive for 2009 H1N1**	21	121^f^

a The middle column represents the complete dataset of HCMC Health Services reports and HTD's RT-PCR results. The last available report includes data on passengers arriving before 13:00 on 9 July 2009. The middle column indicates the number of patients used for epidemiological analysis (Figure 2), and the right column indicates the number of patients from whom data were available for virological analysis (Figures 3 and 4).

b Estimate of 760,000 passengers based on an average of 8,500 daily international arrivals into Vietnam between 27 April and 9 July 2009.

c Difficult to estimate as definition of “epidemic countries” was changing rapidly at this time.

d Thirteen patients with dates of arrival missing were admitted between 9 July and 12 July 2009. They could have landed in HCMC before or after the 9 July 13:00-cutoff time stated in the last HCMC Health Services report.

e 192 of these patients were included in the clinical and virological analysis.

f 97 of these patients were included in the clinical and virological analysis.

A summary of these 619 individuals documented by the HCMC Health Services and HTD from 9 May through 24 July 2009 is presented in Figure 2. The epidemic in Vietnam has subsequently continued with 10,568 confirmed cases by 28 October 2009, after which either the epidemic or case confirmations, or both, slowed down; 53 deaths and 11,104 cases of 2009 H1N1 were confirmed as of 28 December 2009. Figure 2 is presented similarly to a stacked bar graph, such that the height of each colored area represents the number of individuals with a given infection status at each point in time. We estimated the numbers of infectious individuals circulating in the community (red area) on the basis of the self-reported date of illness onset, and the assumption that patients were infectious from onset of illness. Although human influenza infections typically exhibit a presymptomatic infectious period of 1–2 d [10], we did not include this feature in our epidemiological analysis as we had almost no data on the presymptomatic period in our patient group; an individual is counted as potentially infectious if he is symptomatic or PCR-positive. During the first 9 wk of the epidemic, 79.0% of total PCR-positive case days were spent in isolation (95% QID 77.9%–79.9%), and 59.9% of PCR-positive case days (95% QID 58.9%–61.1%) were spent under isolation and treatment, assuming equal infectiousness for each day of illness or PCR positivity, corresponding approximately to a 2-fold or higher reduction of the infectious capability of these individuals, depending on the presymptomatic infectious period. The estimate of the proportion of infectious time spent under isolation is an upper bound, since undetected cases were not isolated. The outbreak response was more effective against travelers than residents, presumably because of active screening at the airport; travelers spent 10.1% of their potential infectious time circulating in the community (and possibly transmitting) as opposed to residents who spent 42.2% of their potentially infectious days circulating in the community. As not all patients with symptoms self-report or are intercepted, and as not all patients with mild respiratory illness are tested, the 321 patients described here are an underestimation of the total burden of infection and community transmission during this time.

**Figure 2:**
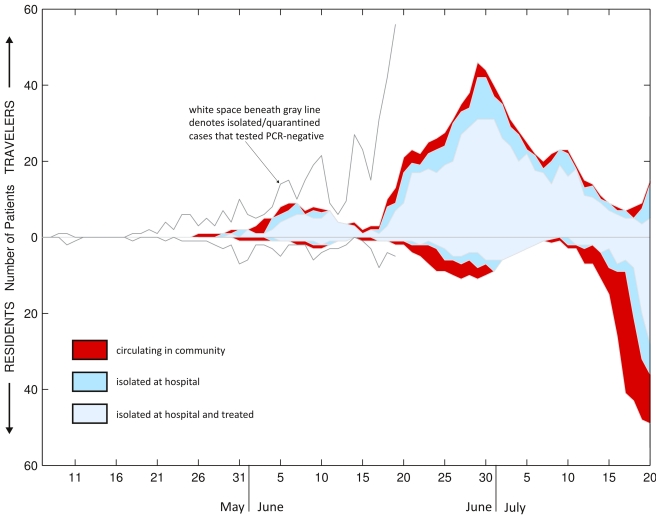
Status of confirmed new cases in HCMC. 321 PCR-confirmed 2009 H1N1 cases and 298 PCR-negative suspected 2009 H1N1 cases admitted to hospitals in HCMC between early May 2009 and 20 July 2009. All 619 individuals are classified either as travelers (those who recently entered HCMC on a commercial flight from a foreign country) or residents; travelers are shown above the axis and residents below the axis. Graph is organized in a stacked fashion, so that the height of each colored area corresponds to the number of patients of a particular status (e.g., circulating, isolated) on a particular day. Graph is cut off on 20 July 2009 as the data were more sparse after this date.

The basic reproductive number (*R*_0_) of the outbreak in HCMC can be calculated from the 14-d period from 7 July to 20 July 2009, which appears to be the initial exponential increase of community transmission. Poisson regression (number of cases versus time) on these 14 data points gives a rate of exponential increase *r* = 0.289 (95% confidence interval [CI] 0.248–0.333), which gives *R*_0_ = 1.5 (95% CI 1.5–1.6) for a generation interval of 1.9 d [11], *R*_0_ = 1.8 (95% CI 1.6–1.9) for a generation interval of 2.6 d [12], and *R*_0_ = 2.0 (95% CI 1.9–2.2) for a generation interval of 3.6 d [13]. *R*_0_ values here are computed using the equation *R*_0_ = 1+*rT*_c_, which assumes an exponential distribution for the generation time *T*_c_ [14]. Confidence intervals must be interpreted with caution as the 14 data points entered into the regression analysis are highly nonindependent. This *R*_0_ estimate for HCMC is in the range of estimates obtained from other densely populated areas for the current pandemic [11],[15], but uncertainty whether the reporting process changed during that period, the small number of data points, and our lack of an endogenous generation time estimate mean that much work remains to be done to understand the basic reproductive number of 2009 H1N1 in Vietnam.

The first case of sporadic community transmission was reported on 5 June 2009, eventually followed by two large outbreaks in schools in the third week of July. Community transmission probably became fully established by mid-July, as indicated by the growing numbers of patients presenting to outpatient clinics in the city.

### Clinical and Virological Analysis

During the first 2 mo of pandemic transmission, 2009 H1N1 diagnostics at HTD were performed on 851 patients, comprising 1,537 individual respiratory specimens and 31 rectal swabs. Of these 851 patients, 292 (34%) were confirmed positive with 2009 H1N1 and are included in the present summary: 195 infected patients consisted of intercepted travelers transferred to HTD for follow-up, and 97 were patients diagnosed through the HTD outpatient clinic or through contact tracing of community outbreaks. Results of molecular analysis and viral culture are presented in Figure S1. Among the cohort of 292, the median age was 26 y (range, 1–72); 193 were men. This discrepancy of male versus female was caused by an outbreak in a secondary school for boys (*n* = 50). Fever was present in 96% (*n* = 281), cough in 59% (171), runny nose in 17% (49), sore throat in 23% (68), and diarrhea in 2% (5). Influenza-like illness (fever and respiratory symptoms [ILI]) was noted in 61% (179). Average duration of fever on presentation was 2 d. After 24 h, 78% (*n* = 228) of patients had a normal temperature. None of the patients experienced pneumonia or severe outcomes. One patient had evidence of infiltration on chest X-ray examination, and all others (*n* = 291) were normal. There was no marked skewing of the age distribution of cases or any apparent correlation of clinical or virological markers by age group. A substantial percentage of patients (47%, 135/290) presented with mild-to-moderate lymphopenia (<1,300/µl). The number of lymphocytes depended inversely on log Ct value infVA RT-PCR, a surrogate measure of viral load (*p*<0.005, linear regression).

**Figure 3:**
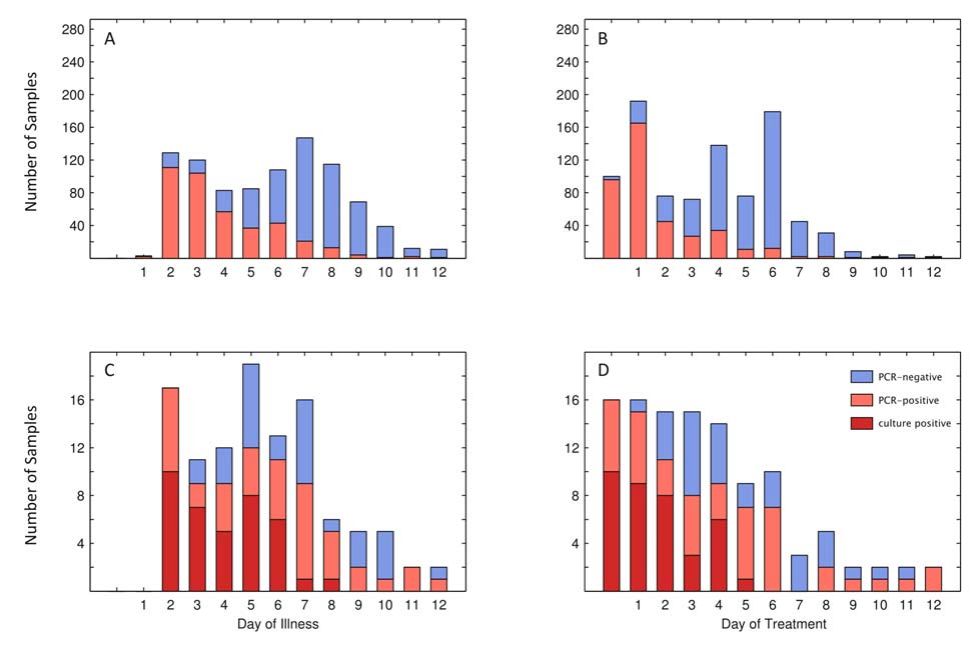
RT-PCR and culture results related to day of illness or treatment. (A and B) PCR status for 932 individual samples by day of illness (A) and day of treatment (B), with the vertical axis extending to 292, the number of patients from whom samples were taken. (C and D) 108 (C) and 111 samples (D) of a total of 115 with which viral culture was attempted. Day one of treatment is the day of treatment initiation. Day zero of treatment is 1 d before treatment is initiated. Three culture-positive samples were PCR-negative: two taken on fourth day of illness (second day of treatment), and one taken on sixth day of illness (fourth day of treatment).

Figure 3 presents a summary of the PCR results from respiratory samples in relation to day of illness and day of treatment. At day 5 of illness, >50% (48/85) of samples taken were PCR negative, and at day 9 of illness >90% were negative (Figure 3A). Similarly, at day 3 of treatment >50% (45/72) of samples were PCR negative, and 1 d following completion of the 5-d oseltamivir course >90% (167/179) were negative (Figure 3B). These figures are biased because of different sampling schedules, and because patients who remained positive were sampled daily, thus causing a bias towards positivity on “late” sampling days. 14 patients were still PCR-positive 1 d after finishing a 5-d course of oseltamivir. Prolonged PCR-positivity did not appear to be correlated with disease severity as no patients had a complicated course of disease, and the median time-to-fever clearance was 48 h in both patients who were still positive at day 5 of treatment or later (*n* = 25) and in patients who were PCR negative at day 5 or earlier (*n* = 180). All rectal swabs were PCR negative (*n* = 31).

Culture isolation was attempted on a total of 115 PCR-positive respiratory specimens representing serial samples from 33 patients. Attempts were especially focused on patients whose PCR results remained positive after treatment. Isolations were successful from 20 of 33 patients (overall recovery 61%), and cultured virus was obtained from 38 of the 115 respiratory specimens (33%) (see Figure 3C and 3D). Culture positivity among PCR positive samples decreased as day of illness or treatment progressed. No isolates were recovered from 21 (11 of which were PCR positive) specimens collected after day 8 of illness or from 29 (16 of which were PCR positive) specimens collected after day 5 of oseltamivir treatment. Attempts to quantify live virus directly from respiratory swabs (days 1 and 2 postadmission) by TCID_50_ or plaque assays were unsuccessful, suggesting that levels of virus shedding were below the limit of detection of our TCID_50_ and plaque assays (100 plaque-forming units [PFU]/ml).

**Figure 4:**
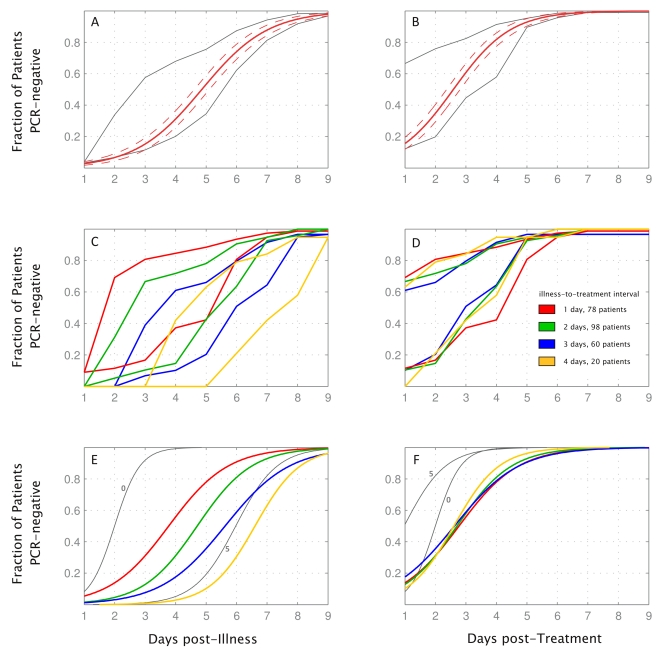
Per patient analysis of RT-PCR results shown by day of illness and day of treatment. Time to PCR negativity and its dependence on illness-to-treatment interval. (A) Gray lines show the minimum and maximum number of patients who were PCR-negative after a certain number of days of illness, on the basis of patients' last positive PCR result and first negative PCR result, which could be separated by a gap of as many as 4 d. Red line shows the ML-fit (see Methods) of time to PCR negativity, and dashed lines are 95% confidence bands. (B) as (A), related to days of treatment. (C, D) Minimum and maximum durations of PCR positivity for patient subgroups corresponding to the length of illness-to-treatment interval. (E, F) ML-curves describing time to PCR-negativity for patient subgroups. Curves for patients who started treatment on the day of illness onset (illness-to-treatment interval = 0, 11 patients), and patients who started treatment 5 d postillness (illness-to-treatment interval = 5, 10 patients) are shown in gray as they differ qualitatively from the other four curves. Legend in (D) applies to (C–F). Data from 278 patients with both negative and positive PCRs were used to make these graphs.

Figure 4A and 4B shows the maximum and the minimum number of days of PCR-positivity in our patient set on the basis of last PCR-positive day and first PCR-negative day for 278 patients for whom these values, as well as day of treatment initiation, were available; the inferred maximum-likelihood curve of PCR-positivity is shown in red. More than 50% of patients had their last positive RT-PCR by day 3 of illness, and their first negative by day 7 (>90% by days 7 and 9, respectively). Counting from the first day of treatment, >50% of patients had their last positive sample by day 2 of treatment and their first negative by day 5 (>90% by days 5 and 7, respectively). Because patients presented at HTD with different illness histories, we were able to partially analyze the effect of timing of treatment initiation by grouping patients according to their illness-to-treatment intervals (first day of treatment minus day of illness onset). For patients with illness-to-treatment intervals of 1 to 4 d, PCR negativity was primarily determined by day of treatment rather than day of illness. In contrast, patients with a longer interval cleared virus earlier during the course of treatment, possibly related to natural course of illness and immune response (see Figure 4C–4F).

According to the maximum-likelihood curves in Figure 4A, 4B, 4E, and 4F, median time to PCR negativity was 4.9 d after illness onset (95% CI 4.6–5.1) and 2.6 d after treatment initiation (95% CI 2.4–2.8). For patients who started treatment the same day as illness onset, median time to PCR negativity was 2.0 d after start of treatment (95% CI 1.5–2.5). For patients who started treatment after illness onset, median times to PCR negativity post-treatment were 2.8 d (95% CI 2.3–3.2), 2.7 d (95% CI 2.3–3.1), 2.6 d (95% CI 2.1–3.1), and 2.6 d (95% CI 1.9–3.3), for illness-to-treatment intervals of 1, 2, 3, and 4 d, respectively.

Samples from 33 patients (*n* = 50) were screened for the presence of the oseltamivir resistance–associated H275Y mutation in the neuraminidase gene by real time RT-PCR. Selection of patients for screening was based on prolonged PCR-positivity (>5 d), with the caveat that only specimens with significant viral RNA (Ct value of 38 or less, as determined by InfVA RT-PCR) were chosen for screening. Phenotypic screening for NAI resistance by IC_50_ assay was performed on selected H1N1 isolates (*n* = 23 specimens, representing 16 patients), and did not yield any evidence for oseltamivir resistance. To date, samples from three patients (hospitalized after the described patient group of 292 patients) have tested positive for the H275Y mutation by RT-PCR and complete sequencing of the NA gene. Specimens collected on admission showed only wild-type virus; the H275Y mutation was present in samples taken on or after day 5 of treatment only. The clinical course of illness in two patients was unremarkable. One patient, a 3-y-old child, required admission to an intensive care unit for respiratory support but made a full recovery within 10 d. No association between emergence of resistance and deterioration of symptoms were noted.

## Discussion

In the 3 mo from 27 April to 24 July 2009, 760,000 passengers who entered HCMC on international flights were screened for 2009 H1N1 influenza: 0.15% were intercepted, 200 had a positive 2009 H1N1 RT-PCR. An additional 121 out of 169 nontravelers tested positive after self-reporting or contact tracing. These 321 patients were isolated and treated, and spent 79% of their PCR-positive days in isolation or under both treatment and isolation (59.9%). Most patients had mild disease and none experienced pneumonia or a severe outcome. Viral clearance was similar among patient groups with different illness-to-treatment intervals, with an estimated median clearance time of between 2.6 and 2.8 d after treatment start for intervals of 1–4 d, and 2.0 d when treatment was started on the first day of illness.

### Effectiveness of Isolation Measures

During the documented period of the containment phase (27 April to 9 July 2009) of the epidemic in HCMC, a high percentage (16%–17%) of intercepted travelers tested positive by RT-PCR for 2009 H1N1 influenza. This number reflects the high levels of global transmission of the novel pandemic virus and is in the range of the typical detection of seasonal influenza in influenza-like illness surveillance programs in the region, usually between 12% and 25% [16],[17]. These levels are, however, not directly comparable as these surveillance programs use more stringent definitions than airport screenings. The 200 total positive cases through 24 July 2009 suggests that more than three symptomatically infected individuals were coming in per day during the 60-d period from 26 May to 24 July 2009, and that roughly 40 presymptomatic, and hence undetectable, individuals would have arrived in HCMC during the same time period (assuming a 1-d incubation period, a 5-d symptomatic period, and that the 200 positive cases were intercepted during the symptomatic period). The epidemic in HCMC was clearly not containable, a conclusion easily inferred from previous mathematical analyses [18]–[20].

Despite the long odds against containment, our analysis indicates that for the 321 mildly symptomatic to moderately ill cases identified in HCMC between 31 May and 24 July 2009—a patient group that represents at least 76% of the documented cases in HCMC at the time—the majority of PCR-positive days were spent in isolation (79.0%) or under both treatment and isolation (59.9%). The containment program of screening, isolating, and treating suspected cases probably had a notable impact on the outbreak's *R*_0_ during the initial weeks, although this impact cannot be assessed quantitatively without knowing the degree of viral circulation among asymptomatic patients or the health-seeking behavior of individuals with respiratory symptoms. Reed et al. [21] suggested that for every reported case in the United States, 79 cases were unreported because of lack of symptoms, care-seeking, testing, or test sensitivity; 33 unreported cases for every reported case were estimated from the same data in the early phase of the epidemic. Even during this early phase in the United States, the virus was probably already established in the community. In Vietnam, on the other hand, no imported cases were detected during airport screening from 27 April to 25 May 2009, and given the active case finding employed in HCMC during the initial response, it is not likely that undetected community transmission at such a scale (multiplier of 33, implying ∼10,000 true cases) was occurring in Vietnam in May or June 2009.

In two European experiences of the early phase of the pandemic, 97% of all confirmed cases (Germany) and 88% of nontraveler confirmed cases (The Netherlands) from active case finding were symptomatic. In The Netherlands, 75 d after the first imported case, no cases of 2009 H1N1 had been detected in the sentinel surveillance system, indicating that a significant amount of asymptomatic community transmission was unlikely during that time [22],[23].

An additional complication when considering a patient group composed of travelers and residents is that we do not know how, once infected, the individual threshold for self-reporting to an outpatient clinic relates to the chance of being intercepted with symptoms of influenza-like illness at an airport screening, and how these differences may bias our interpretations of the effectiveness of control measures. Although we are not able to asses this bias or the health-seeking behaviors of individuals in HCMC, we are currently following a cohort of healthy individuals to measure the degree of asymptomatic 2009 H1N1 circulation. If the asymptomatic fraction turns out to be small or negligible, control measures may have delayed sustainable community transmission. If the asymptomatic fraction is large, control measures likely had no effect.

The intervention strategies put in place in HCMC—airport screening, isolation, and treatment—shortened the duration of viral shedding for each detected patient (Figure 4D, 4F, and [24]), shortened the amount of time each detected patient was circulating in the community, and increased patients' likelihood of hygienic behavior and self-reporting if they had influenza-like symptoms (17% of incoming international flights were given announcements suggesting self-quarantine, mask wearing, and guidelines for monitoring personal health). Certainly, the costs and benefits of airport screenings must be evaluated in light of the relative risks of disease introduction as well as the opportunity cost of concentrating public health resources on slowing the inevitable importation of one disease. As costs of the containment strategy are unknown, this is difficult to evaluate. It is important to note that these data are observational and therefore cannot prove whether the containment efforts delayed community transmission in Vietnam.

### Clinical and Virological Features

Overall, the observed clinical and virological features of 2009 H1N1 influenza in Vietnam confirm the patterns of mild disease observed in other affected countries [25],[26]. There has been much interest in estimating viral shedding durations for the new influenza variant, as these parameters are critical for determining recommended periods of (self)-isolation, and are key to modeling transmission dynamics. In our oseltamivir-treated patient group, we found the time to PCR negativity for the majority of patients was between 3 and 7 d of illness, and between 1 and 3 d after treatment initiation. Only 14 patients (4.8%) had a positive PCR 1 d after completing a 5-d course of oseltamivir. These results are comparable to earlier work on oseltamivir treatment in seasonal influenza [24],[27]. Whitley et al. [27] demonstrated clearance of viable virus after 4 d of treatment in 45% (42/93) of children under 12 y, versus 31% (33/105) on placebo, and full culture negativity by day 6 in both treated and untreated children. Similarly, in a randomized controlled study of experimentally infected healthy volunteers infected with seasonal H1N1, Hayden et al. [24] showed a median duration of viral shedding of 2.4 d (1.4–2.5 d) in the oseltamivir-treated groups (*n* = 56), and 4.5 d (1.6–5.4) in the placebo group (*n* = 13). For the 2009 H1N1 virus, randomized controlled trials have yet to be conducted. However, data emerging from descriptive studies suggests similar trends. One report of untreated patients (*n* = 44) in Canada found 43% of patients remained PCR positive until day 8 postonset [28],[29], and a study from Singapore (*n* = 73) indicated a median shedding time of 6 d postonset, with 47% still being PCR positive at day 7 [30].

Our data indicate that for illness-to-treatment intervals of 1–4 d, viral RNA clearance is determined by the duration of treatment and not by the duration of illness. These data indicate that oseltamivir provides a consistent shortening in the total duration of viral shedding when administered at least during the first 4 d of illness in uncomplicated 2009 H1N1. When given later, viral shedding times in this infection appear to resemble the natural course of uncomplicated, untreated seasonal and pandemic 2009 H1N1 illness [24],[28],[31],[32]. Patients in this study were not randomized, and we cannot rule out any bias in the composition of our patient population through, for example, the influence of symptom severity on health-seeking behavior of outpatients or willingness or ability of infected travelers to board a plane.

Among our patient samples, we were unable to culture virus from samples taken after 5 d of treatment with prolonged PCR-positivity (Figure 3), suggesting that although patients may harbor detectable viral RNA in mucosa, this result may reflect shedding of replication-incompetent virus. Our results on low concentrations of culturable virus are supported by the work of Panning et al. [33] indicating low viral loads in H1N1 specimens (median of 10^4.6^ viral RNA copies/ml). Witkop et al. [32] reported a relatively high percentages of culture positivity among patients in an air force academy outbreak: 41% (9/22) on day 5 and 24% (7/29) on day 7 of illness compared to 36% (7/19) and 6% (1/16) among our patients, respectively. Although the percentage of treated patients among their culture positives was not reported, it is notable that their percentages are higher than ours because we selectively attempted culture on samples from patients shedding RNA for a relatively longer time.

Median estimated viral clearance time in our population was 4.9 d, as compared to 6 d in a recent paper from China describing 426 mild to moderately ill patients infected with 2009 H1N1 [26]. The age distribution among both patient groups was similar. The observed difference in shedding times may be explained by the fact that Cao et al. used the first day of PCR negativity to calculate shedding time (as sampling frequency was not specified, we cannot be sure of this), whereas we estimated shedding time from all 292 patients including data from 50 patients sampled daily. Another explanation may be the fact that 82% of patients described by Cao et al. were treated with oseltamivir, 60% of all patients within 2 d of illness, whereas in our study 100% of patients received oseltamivir and 64% within 2 d. Other authors have reported longer viral shedding times in younger children infected with 2009 H1N1: a median of 8 d from onset of illness for children under 5 y, 6 d for children aged 5–9 y, and 5 d for children aged 10 y or older [31]. No significant differences in viral clearance times between different age groups were found in our dataset, but our dataset only included five children aged 10 y or younger.

Selection of resistant viruses during treatment with oseltamivir was reported to occur at a frequency of 18% (9/50) among H3N2 infected children [34]. Selection of mutants carrying the H275Y mutation during treatment has been described in 0.5% (*n* = 1/150, Influenza A) [35], 4% (*n* = 2/54, seasonal H1N1) [36], and 27% (*n* = 3/11, seasonal H1N1) [37] of treated patients. In our patients, selection of resistant virus did not appear to be a common event: among the 33 longest shedders no H275Y mutant virus was found. We have previously reported on three patients in whom this mutation was selected during treatment. These patients were isolated in our hospital later in August and September 2009 [3].

### Perspectives

Our data provide insights regarding the efficacy of oseltamivir treatment in 2009 H1N1 infection. Our study of the situation in HCMC during the beginning of this outbreak in southern Vietnam suggests that strict containment measures may have reduced community exposure of infected patients, which may have delayed onset of community-based transmission; however they did not prevent the eventual establishment and widespread circulation of pandemic influenza in Vietnam. Failure of containment measures was undoubtedly also due to substantial numbers of imported cases, both symptomatic and asymptomatic, that inevitably escaped detection at airport screenings.

Our dataset is neither complete nor comprehensive, but establishing a systematic and comprehensive sampling scheme and collecting a complete dataset requires time to obtain ethical approval, which was impossible given the rapid response that was required for this unexpected event. We have, however, begun a descriptive clinical trial on oseltamivir treatment of 2009 H1N1 patients starting 12 August 2009, which includes systematic daily sampling using RT-PCR, viral culture, and pharmacokinetic analysis (NCT00985582). Additional future studies to improve understanding of post-pandemic influenza dynamics should focus on demographic shifts in infection patterns [38], the accumulation of herd immunity and the generation of the first escape mutants [39], the risks of the human–animal interface in regions experiencing high levels of endemic transmission [40], competition and displacements patterns between 2009 H1N1 and seasonal H1N1 and H3N2 viruses, and potential virulence changes in the new virus.

## Supporting Information

Figure S1 RT-PCR and culture results for 2009 H1N1 confirmed patients per day of illness. X indicates no PCR was done on that day, + indicates a positive result, − indicates a negative result. Fills indicate if culture was done, green indicates culture was negative, orange indicates culture was positive. Thick black borders surround the days that pateints received oseltamivir, red borders indicate zanamivir treatment.
(0.14 MB XLS)

## Abbreviations

CI: confidence interval
HCMC: Ho Chi Minh City
HTD: Hospital for Tropical Diseases
InfVA: influenza A
QID: quantiles from imputed data
RT: reverse transcription
swInfVA: swine influenza A
